# Potential use of deep learning techniques for postmortem imaging

**DOI:** 10.1007/s12024-020-00307-3

**Published:** 2020-09-29

**Authors:** Akos Dobay, Jonathan Ford, Summer Decker, Garyfalia Ampanozi, Sabine Franckenberg, Raffael Affolter, Till Sieberth, Lars C. Ebert

**Affiliations:** 1 Zurich Institute of Forensic Medicine, University of Zurich, Winterthurerstrasse 190/52, CH-8057 Zurich, Switzerland; 2 Department of Evolutionary Biology and Environmental Studies, University of Zurich, Winterthurerstrasse 190, CH-8057 Zurich, Switzerland; 3 Department of Radiology, University of South Florida Morsani College of Medicine, 2 Tampa General Circle STC 6097, Tampa, FL 33606 USA; 4 Institute of Diagnostic and Interventional Radiology, University Hospital Zurich, Raemistrasse 100, 8091 Zurich, Switzerland

**Keywords:** Deep learning, Convolutional neural networks, Computed tomography, Forensic sciences, PMCT

## Abstract

The use of postmortem computed tomography in forensic medicine, in addition to conventional autopsy, is now a standard procedure in several countries. However, the large number of cases, the large amount of data, and the lack of postmortem radiology experts have pushed researchers to develop solutions that are able to automate diagnosis by applying deep learning techniques to postmortem computed tomography images. While deep learning techniques require a good understanding of image analysis and mathematical optimization, the goal of this review was to provide to the community of postmortem radiology experts the key concepts needed to assess the potential of such techniques and how they could impact their work.

## Introduction

Postmortem computed tomography (PMCT) has been shown to be a valuable tool in forensic medicine. For instance, a meta-analysis by Ampanozi et al. concluded that PMCT is reliable in detecting skeletal fractures [1]. Furthermore, PMCT angiography helps to add soft tissue contrast to these images and is highly sensitive to soft tissue and organ findings. It is, therefore, well suited for the detection of hemorrhages. Depending on the jurisdiction, PMCT and postmortem computed tomography angiography (PMCTA) are being used as triage tools and/or as additional investigation methods to complement autopsy [1].

PMCT scans can consist of well over 10,000 single images. Although in practice only forensically relevant findings need to be analyzed, this can amount to a substantial workload on forensic pathologists. In clinical radiology, similar issues regarding workload exist [2], and machine learning approaches are being developed to address this issue [3, 4]. For these reasons, automated analysis of PMCT images was one of the research focuses identified by the first postmortem radiology and imaging research summit, which was organized by the International Society of Forensic Radiology and Imaging, the International Association of Forensic Radiographers, the National Institute of Justice of the United States of America, and the Netherlands Forensic Institute [5]. The aim of this article is to present the technical background and underlying concepts of deep learning and to highlight its potential use for analyzing PMCT in postmortem radiology based on experiences from clinical radiology.

Image analysis for postmortem computed tomography (PMCT) differs significantly from image analysis in clinical radiology. The clinical radiologist often has a defined focus for an examination, and only a limited area is scanned to minimize the radiation dose and scanning time for the patient. In postmortem radiology, the entire body is captured at high resolution to ensure that every pathology, anatomical anomaly and foreign body is documented. Furthermore, the image quality has to be sufficient for identification, visualization and complex reconstructions [6]. As the radiation dose does not need to be considered, the scan protocol is optimized for image quality, which leads to more detailed images and therefore larger datasets. In clinical radiology, the primary aim is to exclude or diagnose a pathology or injury correctly to choose the appropriate treatment. In postmortem radiology, determining the cause and manner of death is the primary focus of attention. In addition, PMCT data can be used in the reconstruction of the sequence of events, such as traffic accidents or homicides [7, 8]. In unknown persons, PMCT data are used for identification via comparison with images obtained before death (for example dental radiographs [9]).

Additionally, exclusion of findings can be equally important. This has an impact on how the PMCT images are obtained and analyzed. The scan protocols usually document the entire body, with additional higher resolution scans for the thorax, abdomen, head and teeth, which can add up to 10,000 single images or more [10]. It is common practice in many countries that the same extensive protocol is used, independent of the expected findings. This means that the postmortem radiology expert always has to assess the entire CT dataset for forensically relevant pathologies, injuries and the presence of foreign bodies [11]. In some cases, segmentation is also required to estimate volumes and weights or as a basis for advanced visualizations or 3D prints to be presented in a court. Due to the number of images available and depending on the complexity of the case, reading can take hours. Organ segmentation (the act of isolating relevant structures) can be equally time-consuming. A lack of trained postmortem radiology experts, in conjunction with the costs of a long reading process, may sometimes limit the use of radiology in death investigations. To increase the quality of the image analysis and decrease the costs, new tools tailored to the specific needs of postmortem radiology experts are required. This includes but is not limited to automatic organ segmentation and weight estimation and injury and fracture detection, as well as foreign body detection and identification.

As image data are inherently digital in nature, computer methods are an obvious source for tool creation. Using conventional image processing methods, the developer of an algorithm has to identify relevant structures, choose the right set of image processing techniques and fine-tune the algorithm until the selected feature is detected correctly. The problems with this approach are that developing and fine-tuning such an algorithm is time consuming and requires expert knowledge with respect to the structure to be identified as well as to which image processing techniques are available. Algorithms based on conventional image processing can have low robustness for images that have great variance or contrast gradients, such as in magnetic resonance imaging (MRI) images or noisy CT scans. One way to overcome these issues is the use of specialized techniques from artificial intelligence (AI), so-called deep learning techniques [12]. In this article, we aim to give researchers in the field of postmortem radiology a starting point to implement deep learning techniques themselves. We present the technical background and underlying concepts of deep learning and highlight its potential use in postmortem radiology for analyzing PMCT data based on experiences from clinical radiology.

## Understanding deep learning techniques

**Fig. 1:**
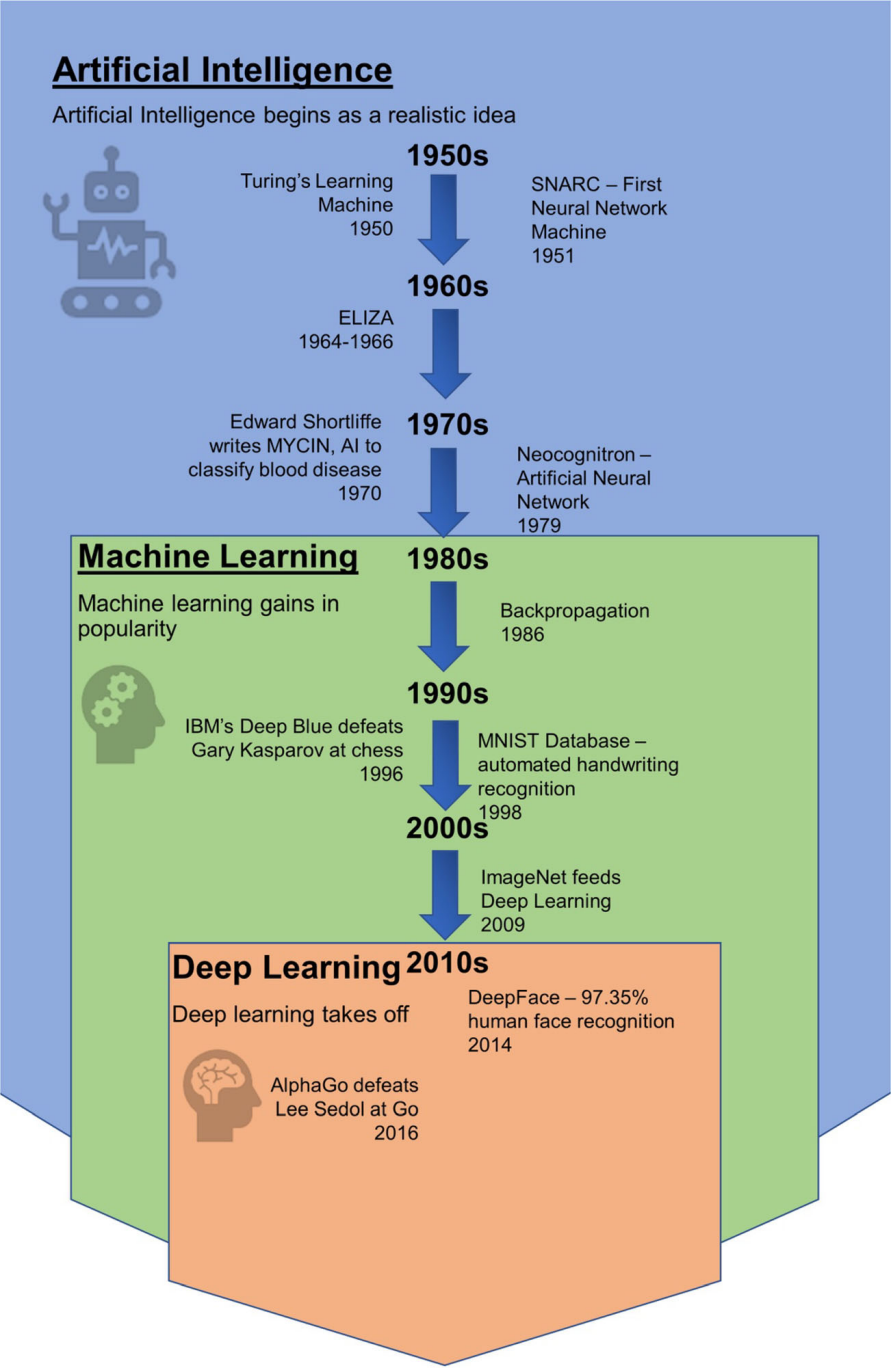
Timeline of deep learning milestones

AI is a subcategory of computer science that tries to mimic human intelligence to solve complex problems. Examples of classical AI systems are early chess computers and handwriting recognition. While many AI techniques are purely algorithmic, a subset of AI called machine learning (ML) addresses a more data-driven approach. With ML, algorithms analyze sample data to adjust their behavior, thus learning from the data rather than just following a predefined procedure. An example of ML is spam filtering in email programs, which performs statistical analyses of emails that are marked as spam to categorize incoming emails. Finally, deep learning (DL), which is a type of machine learning, uses artificial neural networks (ANNs) to analyze the data. ANNs mimic, in a simplified manner, how nerve cells function and communicate with each other to express complex behavior from relatively simple building blocks. The basic building block of an ANN is the aptly named artificial neuron. Such a neuron consists of multiple weighted inputs that are summed to produce an output that is then analyzed. A timeline of major milestones in AI can be seen in Fig. 1 [13–22].

**Fig. 2:**
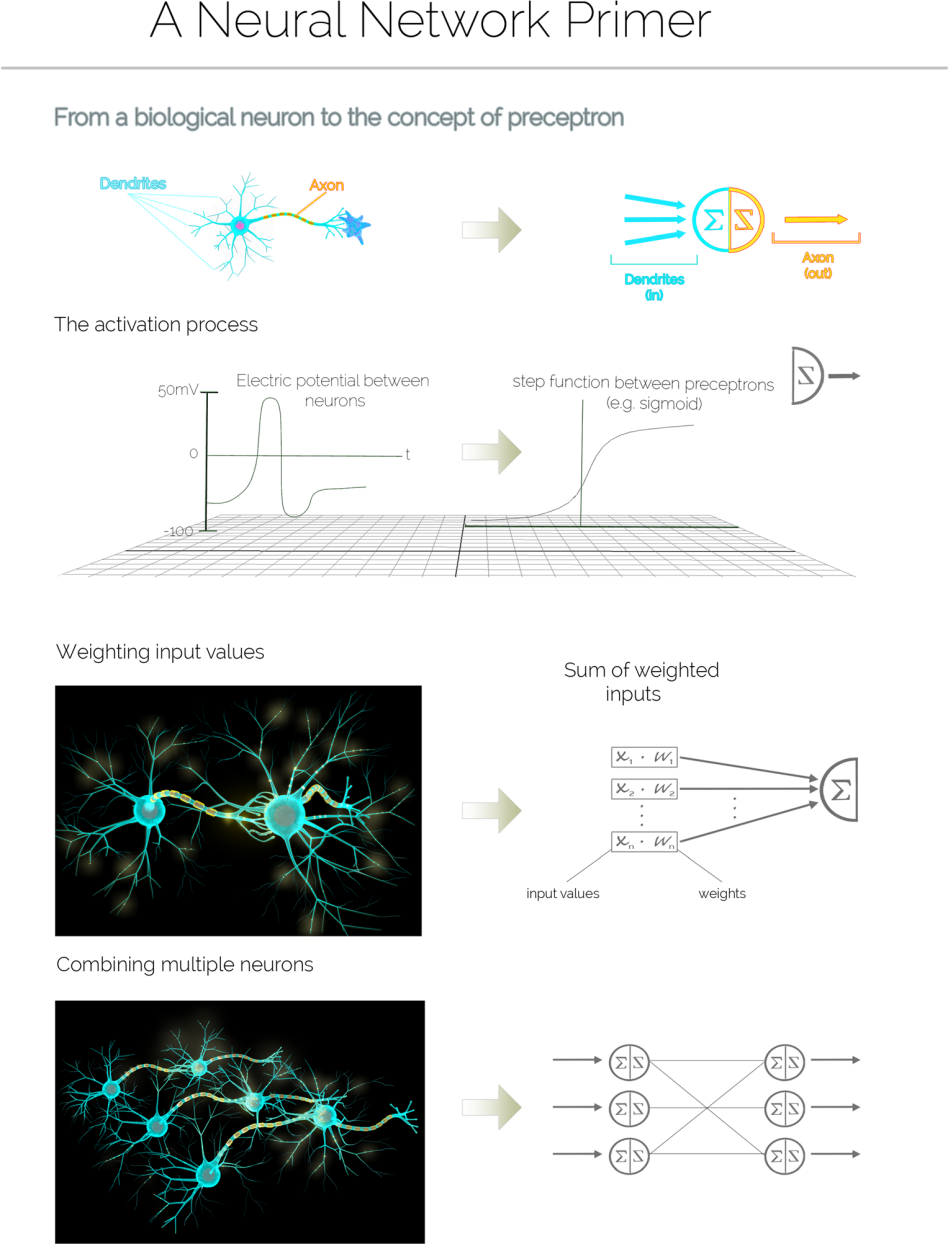
Basic concepts of neural networks. The building block of a neural network is the perceptron: a mathematical abstraction simulating the main function of a neuron. The signal received from other dendrites (inputs) is further propagated through the axon (output) if the overall stimulus that arrives at the cell body (perceptron) is large enough to trigger an action potential (step function/sigmoid). Each input value is modulated by a weight, and the action potential is the weighted sum of the inputs. In a neural network, each perceptron is called a node, and nodes are organized into layers. Each node in a layer can be connected to all other nodes in the subsequent layer. This architecture is repeated until the output layer contains the various classes of interest

The idea of artificial neurons can be traced back to 1943, when Warren McCulloch and Walter Pitts proposed modeling the nervous system as a network of logical units able to integrate over several inputs by summing them up and producing an output. Frank Rosenblatt developed this idea further in his concept of the perceptron [23, 24]. Interconnected systems of perceptrons evolved into what we now call artificial neural networks (ANNs) or simply neural networks (NNs): a class of algorithms with the goal of classifying a set of data into categories. The architecture of NNs is similar to that of a biological neuron. Dendrites represent separate input channels with their specific weights. The overall input signal is integrated into the cell body. If the accumulated signal exceeds a certain threshold, an output signal is produced. Figure 2 proposes a simple illustration of how the concept of NNs evolved from the nervous system.

**Fig. 3:**
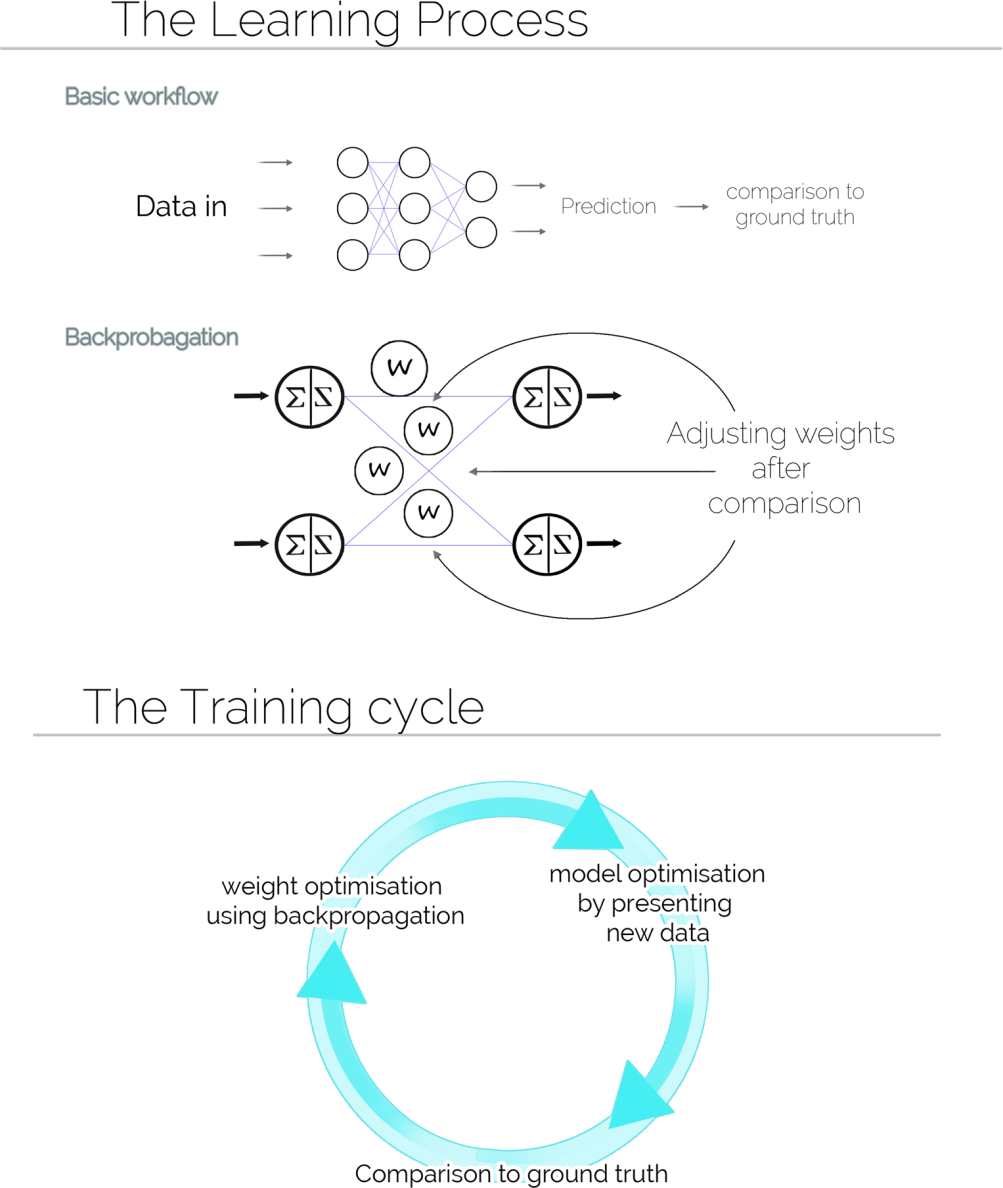
Training a neural network. Through backpropagation, the weights between the nodes can be adjusted. This process uses a stochastic gradient descent by which the output of the neural network is compared to the ground truth. When the difference is smaller than a user-defined threshold, the model is retained. In a new round of comparison, the model is further validated with a new dataset to fine-tune the weights. This process can be repeated multiple times. However, if repeated too often, it can result in overfitting

**Fig. 4:**
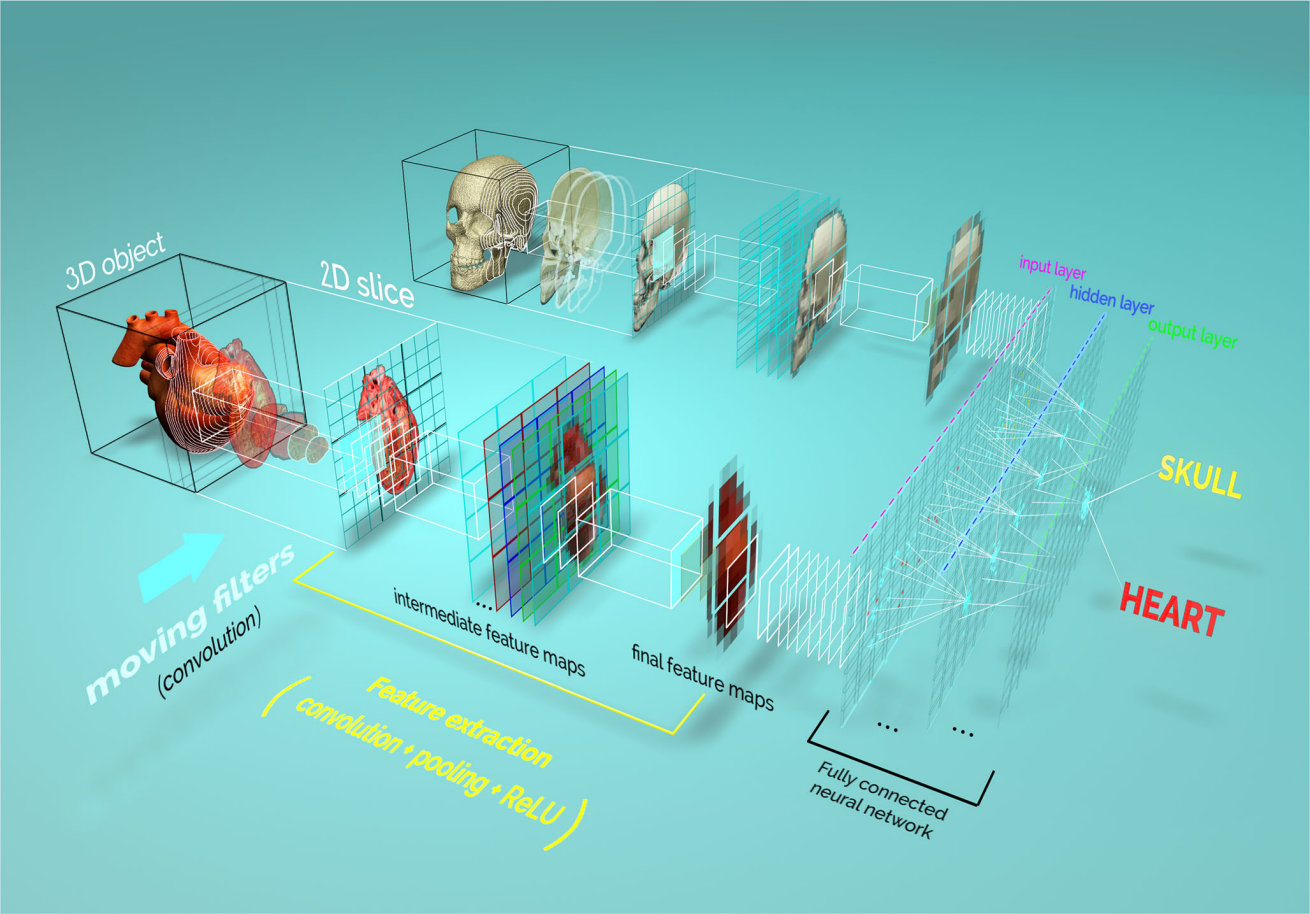
Graphical depiction of convolutional neural networks (CNNs). A real object is reduced to a series of images (2D slices or projections, depending on the region of interest). The images are then presented to the convolutional neural network. First, features, such as edges, ridges, or blobs, are detected using a series of mathematical filters (convolution). Each resulting image is stored in a smaller format (pixel pooling). In addition, a linear rectifier unit (ReLU) is applied to remove all negative pixel values between two convolution operations. The new image, containing an abstract description of the original image, is called a feature map. This process is repeated several times, and the final feature maps are converted into a one-dimensional array (vector) to form the input layer of the fully connected neural network (FCN). This operation is often called flattening. While it is common to have multiple hidden layers between the input and the output layers of the fully connected neural network, the output layer is directly connected to an activation function that gives the likelihood that each category of objects is present in the original image. Once the model has been trained to recognize a specific type of object, e.g., a heart on a CT section scan, the model will give a probabilistic answer to the question: does this image contain a heart?

Mathematically, NNs are constructed using interconnected nodes arranged in layers. Each node of a layer receives inputs from nodes of the previous layer. The combination of layers is called a topology and consists of a fully connected network. Hence, depending on the amount of input signal, some nodes will propagate the signal while others will stop it. Interconnected layers are divided into three groups: the input layer, the output layer and the hidden layers. The input layer is directly connected to the data set, while the output layer can be seen as a real-valued vector. Intermediate layers are called hidden because their values are not directly used for classification. A proper classification relies on the optimal value for each individual weight in the network. Fixing the weights by exploring all possible combinations is unrealistic for large networks. The backpropagation algorithm uses an objective function (cost function) that is constructed with the values of the output layer and the target values. Taking the first derivative of the objective function tells us the direction of its gradient, which is then multiplied by a constant parameter. This discrepancy constitutes the error that we want to minimize. This error is backpropagated to the input layer to adjust the weights. A similar process is used to backpropagate the error for all hidden layers by multiplying the error between each hidden layer together. Finally, a softmax function is used to normalize the value of the output layer to create a probability density function that tells the likelihood associated with each category represented in the output layer. Figure 3 depicts the full cycle of how NNs are trained to obtain a model to be used to classify the input data. The complexity of an NN grows with the complexity of the data. For instance, an image of 512 × 512 pixels (the typical size of a single slice of a PMCT dataset) would need an input layer with 262,144 nodes, one for each pixel in the image, as well as numerous hidden layers for processing them. Optimizing the weights for such NNs was unrealistic with computers in the 1980s. The question was how to reduce the complexity to make it feasible for a computer to classify images. Convolutional neural networks (CNNs) are a solution proposed to reduce network complexity. A graphical depiction of this process can be seen in Fig. 4.

CNNs have existed since the late 1980s [25] and were initially developed for handwritten digit classification. Through convolution, the size of an image is typically reduced to a few thousand pixels. The new image constitutes a feature map of the original image; visually, these two images are different. The convolution applies a square filter that typically has few pixels equivalent in size to the pixels of the original image. In other words, the convolution is a mathematical procedure that uses the pixels in the direct neighborhood of a focal pixel to reinforce the information content. Local features of an object can be reinforced by this means or blurred out. The next step consists of selecting only the pixel with the maximum intensity in a local area and eliminating any negative values. This step is called maximum pooling while the pooled values are passed through a linear rectifier called rectified linear unit (ReLU). Through the pooling action, the image size is reduced. The whole process of filtering, rectifying and pooling the pixels of an original image is repeated several times and with several sets of filters. All the feature maps are combined together into a single array to form the input layer of the NN.

## Medical image analysis

Medical image analysis can be traced back to as early as the 1970s using various mathematical modeling and ruled-based algorithms. In the 1990s, supervised techniques, such as shape models and atlas-based segmentation methods, were used to identify organs and extract features for statistical classification in computer-aided diagnosis. An important revolution in image analysis and image manipulation appeared with the development of graphics processing units (GPUs), dedicated electronic circuits separated from the main processor (CPU) that were intended to specifically accelerate the manipulation of graphic contents. Since Steinkraus and his colleagues [26] have shown the value of GPUs for ML, following the recent development in GPU hardware over multicore CPUs, CNNs have become a popular approach for image processing [27]. Dedicated hardware is now commercially available to perform calculations on large datasets.

CNNs started to gain popularity when the winning team of the 2012 ImageNet challenge used them for their model [28]. ImageNet is a public repository of human-annotated Internet images organized by concept [ref: http://image-net.org]. The ImageNet challenge was created by the ImageNet community to foster innovation in image classification. Since then, numerous studies have been published showing how CNNs, together with other ML techniques, are used to improve medical image analysis, such as that for CT, MRI, positron emission tomography (PET) and radiographics. State-of-the-art results were achieved in mammographic mass classification [29, 30], segmentation of lesions in the brain [31], leak detection in airway tree segmentation [32], lung pattern classification [33], prostate segmentation [34, 35], nodule classification [36, 37], breast cancer metastasis detection in lymph nodes [38, 39], human expert performance in skin lesion classification [40], and bone suppression in radiographics [41]. Most CNN approaches are based on processing 2D images. Medical (CT) data add a level of complexity, as they are 3D in nature and require 3D volumetric segmentation and analysis [42, 43]. Therefore, there is a need to develop CNNs for 3D volumetric data.

## Possible applications of deep learning techniques in PMCT

Previous work done on segmentation of radiological data for abdominal organs has been performed using multi-atlas [44], patch-based [45] and probabilistic atlas methods [46]. More recently, a fully convolutional network (FCN) has been developed for medical image segmentation. The flowchart of the network visually follows a U-shape and has been labeled U-Net [47]. The U-Net architecture was subsequently extended by Çiçek et al. [48]. Larsson, Zhang and Kahl proposed a two-step method, where first the organ is registered using its center of gravity, and then voxelwise binary classification is applied using a CNN [49]. The method has the apparent advantage of delivering more reliable results for organs with high anatomical variability. There is no reference in the literature on automated segmentation of organs using 3D PMCT images. Automated segmentation of organs based on PMCT images could be used, for instance, to estimate organ weight and detect anomalies such as hemorrhagic pericardial effusion [50, 51]. Knowing the exact position of an organ can also help in planning procedures such as CT-guided needle placement [52].

Age estimation based on facial recognition can be traced back to the work of Kwon and Lobo [53]. Since then, several techniques using either geometric ratios of anthropomorphic features or support vector machines on key landmarks have been applied. Works that specifically use DL to extract features were introduced in other studies [54–56].

In mass disaster events, PMCT can help speed up the process of identifying victims [57]. PMCT also allows for sex, age, ethnicity and stature estimation in the investigation of unknown remains [58]. Age estimation techniques utilize a variety of features, such as sternal rib ends, sacroiliac joint morphology, the pubic symphysis and cranial sutures. Additionally, dentition staging and development are highly useful in age assessment of juveniles [59]. Some techniques, such as statistical shape modeling, require 3D models extracted from PMCT data, and these models could be automatically extracted using automated segmentation [60].

In a postmortem examination, the time of death is estimated using several parameters, such as lividity, body stiffness, body temperature [61], presence of insects, environmental factors, and others. With PMCT, additional postmortem changes, such as gas formation inside the body of the deceased, can be assessed [62].

Fracture detection using DL produces reliable results in conventional radiographs obtained at the hospital; Kim and Mac Kinnon combined the Inception (version 3) pretrained model to identify fractures from lateral wrist radiographs [63].

CT scans of the head are not only able to show plain pathologies, such as hemorrhage, tumors and fractures, but also make it possible to derive the sequence of complex fractures and the direction of an inflicted gunshot [64–66]. The use of deep learning for CT scans has been investigated in several studies. For instance, in Chilamkurthy et al., the authors use natural language processing to detect key findings in intracranial hemorrhage [67]. A similar study was performed by Arbabshirani et al., in which the authors used a CNN approach instead [68]. To date, no studies have been published on the use of machine learning for gunshot injuries.

For future research, we propose the following three-step plan of action. Because machine learning requires a large amount of structured data, the first step is to set up appropriate databases, if possible, in a collaborative effort to increase the volume of data. For clinical radiology, similar databases have been developed. The second step is to adapt existing methods from clinical radiology. Because PMCT data can be different from clinical CT data (i.e. due to inner livores or putrefaction), it is required that existing network topologies be retrained with postmortem data. This approach will help research groups in the field of forensic imaging build up their own expertise in machine learning. Finally, new network topologies can be developed that target image processing problems specific to postmortem imaging.

## Conclusion

The use of deep learning techniques to automate CT image analysis can be found in various fields of medicine. Studies reporting successful implementation of machine learning techniques to classify CT images involved applications ranging from fracture detection to the detection of pathologies, such as cancer and skin anomalies. Convolutional neural networks constitute the dominant choice in regard to choosing a deep learning algorithm for medical diagnosis. This can be partly explained by the large number of existing frameworks, such as TensorFlow, Keras or PyTorch. With little knowledge of programming and few lines of code, these frameworks enable researchers to build complex CNNs. Therefore, most of the work relies on collecting enough data to train a model and preprocessing the data to make them compatible with these frameworks. Additional work is also necessary to develop appropriate network architectures and topologies.

Although there is already much literature on the use of deep learning techniques in clinical CT image analysis, there is little on the application of DL in forensic medicine and PMCT to date. Many possible applications in this specific area remain to be investigated.

## Key points

1. Deep learning techniques could help to compensate for the lack of postmortem radiology experts.
2. Numerous studies show how convolutional neural networks improve 2D medical image analysis.
3. 3D volumetric data add another level of complexity for deep learning techniques.
4. Little research on applying deep learning techniques in forensic medicine can be found.
